# Electronic data capture in a rural African setting: evaluating experiences with different systems in Malawi

**DOI:** 10.3402/gha.v7.25878

**Published:** 2014-10-30

**Authors:** Carina King, Jenny Hall, Masford Banda, James Beard, Jon Bird, Peter Kazembe, Ed Fottrell

**Affiliations:** 1 Institute for Global Health, University College London, UK; 2 MaiMwana Project, Mchinji, Malawi; 3 Department of Computer Science, City University London, London, UK; 4 Baylor College of Medicine Children’s Foundation, Lilongwe, Malawi

**Keywords:** mHealth, electronic data capture, Sub-Saharan Africa, MIVA, ODK Collect, CommCare, Pendragon

## Abstract

**Background:**

As hardware for electronic data capture (EDC), such as smartphones or tablets, becomes cheaper and more widely available, the potential for using such hardware as data capture tools in routine healthcare and research is increasing.

**Objective:**

We aim to highlight the advantages and disadvantages of four EDC systems being used simultaneously in rural Malawi: two for Android devices (CommCare and ODK Collect), one for PALM and Windows OS (Pendragon), and a custom-built application for Android (Mobile InterVA – MIVA).

**Design:**

We report on the personal field and development experience of fieldworkers, project managers, and EDC system developers.

**Results:**

Fieldworkers preferred using EDC to paper-based systems, although some struggled with the technology at first. Highlighted features include in-built skip patterns for all systems, and specifically the ‘case’ function that CommCare offers. MIVA as a standalone app required considerably more time and expertise than the other systems to create and could not be customised for our specific research needs; however, it facilitates standardised routine data collection. CommCare and ODK Collect both have user-friendly web-interfaces for form development and good technical support. CommCare requires Internet to build an application and download it to a device, whereas all steps can be done offline with ODK Collect, a desirable feature in low connectivity settings. Pendragon required more complex programming of logic, using a Microsoft Access application, and generally had less technical support. Start-up costs varied between systems, and all were considered more expensive than setting up a paper-based system; however running costs were generally low and therefore thought to be cost-effective over the course of our projects.

**Conclusions:**

EDC offers many opportunities for efficient data collection, but brings some issues requiring consideration when designing a study; the decision of which hardware and software to use should be informed by the aim of data collection, budget, and local circumstances.

Paper-based data collection methods have been standard in research and routine health settings for centuries. Advancements in mobile technology and its widespread availability have promoted the use of ‘electronic data capture’ (EDC). Although research to evaluate impact of mobile health interventions has been growing, publications are lacking on the use of mobile technology as a research instrument. The potential that mobile technology holds compared to a traditional paper-based process is an important area for investigation (1, 2).

EDC has several potential advantages, including: quicker turnaround time from field to analysis; improved data quality; in-built checking and consistency rules; and sophisticated automated skip patterns (1, 2). Additional hardware-dependent features such as Global Positioning System (GPS) and user-independent time-stamps can assist with monitoring work rate and data validation (3).

There are also potential limitations to EDC which need consideration such as data security, connectivity, and the need for field staff that are comfortable using the technology. Members of rural populations, from whom field staff are often recruited, are frequently not experienced with computer technology and may be put off trying to learn (4). This may be particularly relevant if field staff of a particular demographic are needed, although it can be seen as an opportunity for capacity building within communities. Mobile phones are valued items in resource-limited settings (5), which can lend status and respect to the research staff; however, the EDC device or research staff may be targeted for theft when working in the field (1).

As well as the rapid development of hardware, there are several software options available for EDC, and this is a key consideration. To choose an appropriate EDC method, context-specific factors such as infrastructure and the technical capacity of developers and fieldworkers need to be taken into account. As the use of EDC becomes more widespread in settings such as rural Sub-Saharan Africa, more researchers will be faced with choosing an appropriate EDC system.

We describe our experience with four EDC systems used simultaneously in rural Malawi (Table 1), highlighting key considerations for organisations considering EDC.

**Table 1:** Summary of the electronic data capture software and hardware being used in Mchinji district, Malawi

	Software	Hardware	# of users	Project summary (duration)
Pendragon	Commercial, general purpose form design and administration system for Palm and Windows OS	PDA	12	Cross-sectional survey evaluating the impact of a health education radio programme (3 months)
CommCare	Open-source, but commercially supported, system with web-based form design, data management and reporting systems for Android	Android smartphone	44	Prospective cohort investigating pregnancy intention and maternal and neonatal health (18 months) Prospective cohort investigating risks of treatment failure in community-treated pneumonia (9 months)
ODK Collect	Open-source form design, mobile data collection and management system for Android	Android smartphone	8	Verbal autopsy interviews as part of a larger prospective cohort study to evaluate the impact of vaccine introduction on post-neonatal infant mortality (36 months – still on-going)
MIVA	Purpose-built, stand-alone application for Android, to collect and process verbal autopsy data	Android smartphone	8	

OS: Operating System; PDA: personal digital assistant; ODK: Open Data Kit; MIVA: Mobile InterVA (www.interva.net).

## Context, setting, and method

All four EDC systems were used in Mchinji district, central Malawi, for research projects (March 2013 onwards), with a total of 64 devices being used in four different projects (Table 1). Mchinji has an estimated population of 500,000, 80% of whom live in rural communities where mobile phone ownership is approximately 35% (6).

CommCare was used in two prospective cohort research studies. The first investigated the relationships between pregnancy intentions and maternal and neonatal health. The second was investigating risks of treatment failure in community treatment of pneumonia in children. CommCare was chosen specifically for these two projects because of the ‘case’ function which allowed multiple interviews to be reliably linked, as well as the child’s interviews to be linked to the mother’s in the first project.

Pendragon was used in an evaluation of a health education radio programme on health knowledge and behaviours; our organisation already owned the personal digital assistants (PDAs) and given the benefits of EDC, we chose to use these over purchasing new hardware because of a limited budget. This may be a common situation in resource-poor settings, where organisations already own this out-of-date technology, and it is important to know how these fare against newer (more costly) hardware. Fieldworkers using Pendragon and CommCare were recruited from the local communities where they would be working for the duration of the projects. Most did not have experience of fieldwork or EDC technology and were required to have completed at least 4 years of secondary school, providing significant opportunities for capacity building.

ODK Collect and Mobile InterVA (MIVA) (7) were used together in a large-scale evaluation of vaccine introduction on post-neonatal infant mortality, to collect information on cause of death from verbal autopsies (VA). MIVA (which we have included to demonstrate a custom-built application) is a bespoke ‘app’ designed in collaboration with the World Health Organisation (WHO) to meet the pressing need for simpler VA data collection and processing, as a means to increasing the coverage of operational and representative cause of death registration systems (8). The app is built for android devices and is comprised of more than 200 questions, with skip patterns corresponding to the WHO 2012 standard VA tool. We used ODK Collect in conjunction with MIVA, as we wanted to collect additional information on socio-economic and vaccine status. MIVA could not be customised to collect additional information as it is a stand-alone phone application. Fieldworkers for this project were our most senior level of fieldworker, with all having more than 5 years’ experience with the organisation, and had been awarded or were studying for diplomas, mostly in ‘Community and Development’.

We asked all developers and project managers (between one and two) and at least five fieldworkers from each project to comment on their experiences using an open semi-structured questionnaire with regard to: technical support, and cost and ease of development (project managers and developers); and ease of use, data processing, and available features (all). Themes were synthesised from these responses, and added to from extensive personal field and development experience.

## Development considerations

### Ease of development

Development of the stand-alone MIVA application was done using an open-source development environment that could be programmed offline but required specialist programming knowledge and experience. The other EDC tools were developed by non-specialists having no previous experience to programming experts with more than 20 years’ experience. ODK Collect and CommCare both have user-friendly web-interfaces for designing forms and programming simple logic, or can be developed offline by creating a spreadsheet describing the required form and allowing for more complex logic. However, CommCare requires an Internet connection to build the form and download it on to the smartphone. We found this dependence on the internet in a limited-connectivity setting to be a considerable limitation, e.g. updating forms in the field often took several attempts and considerably more time than doing it via USB. As a result, we would preferentially select ODK Collect over CommCare in studies not requiring multiple visits for this reason. Pendragon form design is done in Microsoft Access (requiring Microsoft Windows) and all logic has to be programmed using a proprietary scripting language.

### Technical support

For the open-source products (ODK Collect and CommCare) good support is available on the Internet from both the developers and other users; specifically for CommCare, Dimagi (the product developer), also provides some support with relatively quick responses as standard, and additional support can be purchased for individual projects. For Pendragon, there is a user manual, but limited online support, which was generally not as easy to access as the open-source software. As MIVA is a standardised stand-alone ‘app’ it cannot be modified locally and therefore there is no technical support available beyond the specific user guide.

### Cost

The two main initial costs are for the software and form development, and the hardware. These start-up costs were difficult to quantify for all projects, as some hardware (PDAs) was already available and the person-time spent creating, modifying, and maintaining the EDC tools varied from a few days for a simple form, to months and years for the more complicated systems. The smartphones we purchased all cost around 100 USD, and prices are likely to continue decreasing. Although the start-up costs were considered to be higher than a paper-based system, there is no data entry, or printing and photocopying costs for EDC. In the long term EDC is likely to be cheaper (as skills in form development increase, and hardware costs are written off), and perceived higher data quality makes it justifiable (9).

For on-going costs, because the majority of fieldworkers do not have electricity at home, they were given 7 USD/month to access commercial charging services. Solar chargers, which could be more cost effective in long-term projects and remove issues encountered when using commercial charging services, have been used successfully elsewhere (3). In our use of CommCare, data were uploaded to the server over cell phone Internet, incurring a total estimated cost of 30 USD/month. CommCare has a fee for more than 50 users (1 USD/additional user) or to access premium features (starting at 100 USD/month). Pendragon requires a one-off license fee (250 USD), with an additional fee (50 USD) for subsequent users. Compared to the running costs of a paper-based system, this is considerably less (e.g. for one data entry clerk and 500 multi-page questionnaires a month, the running cost of a paper-based system in our context would be 350 USD/month). The need for data cleaning is considerably reduced because of in-built cleaning rules, saving time and costs by reducing the need for field verification, and manual data checking and correcting.

Finally, there is the replacement of damaged, lost, or stolen hardware, including chargers, SD cards, and the devices themselves. Over 18 months of continuous data collection we have replaced 11 broken chargers, 2 batteries, 3 stolen SD cards, and 4 devices (out of 64), as well as repairing another device, which we did not consider to be unreasonable.

## Implementation considerations

### Ease of use

All fieldworkers preferred EDC to paper-based systems, one commenting that carrying paper-based systems can be tiresome, whereas the EDC is easier to carry, control, and work with. This is an important consideration when field workers in rural areas often cover large geographical areas, mostly by bicycle. The fieldworkers also commented that the technology was well accepted by respondents in the field.

One week’s training was conducted jointly for MIVA and ODK Collect, in which fieldworkers were orientated on the project protocol; and introduced to smartphones and using the EDC tools. All project managers thought that this was enough time, although only half of the fieldworkers agreed. For CommCare both 1 and 2 weeks of training was conducted for different projects, and for Pendragon, 2 weeks training was provided; again all project managers thought this was sufficient time, but one-third of fieldworkers disagreed, even when training was 2 weeks. This may be due to financial (e.g. residential trainings provide meals and money for incidentals) or other benefits (e.g. certificates indicating the amount of training received). Furthermore, despite training sessions including mock-interviews, and in the case of CommCare, field test interviews, these are unlikely to cover every possible scenario or technical issue. As most fieldworkers subsequently faced a challenging interview or technical glitch, this may also explain why they thought more training was needed.

Despite the majority of fieldworkers never having used a smartphone or PDA previously, most became competent quickly, and an initial lack of familiarity does not seem to have been a barrier to adopting an EDC system. However, when using MIVA and ODK Collect simultaneously to capture different pieces of data in a single interview, the majority of fieldworkers found it difficult to switch between these two systems during the interview, even though these were senior fieldworkers. We found the inability to modify the pre-designed ‘app’ to be a major limitation in a research setting and have since decided to create a single form using ODK Collect.

Technical glitches such as forms freezing, forms not appearing, and difficulty in saving were encountered occasionally in all the EDC systems. No smartphone users reported problems with battery life or accessing and using commercial charging services; however more than half the fieldworkers using PDAs reported issues with poor battery life was an issue, unsurprising as the PDAs were second hand at the start of the project.

### Features available

A summary of features available for each EDC system is presented in Table 2. Of particular note was the ‘case’ function in CommCare, allowing collected information from questionnaires to be stored on the smartphone and used in subsequent questionnaires. This allows interviews conducted at different times for the same respondent (e.g. a pregnant woman who is then followed up post-natally) to be linked reliably, and allows information collected in one interview to be used to control routing and validation in subsequent interviews. For the two projects where we had more than one interaction with the same household, CommCare was the only software which provided in-built linking making it the obvious choice.

**Table 2:** Summary of the key features, data transfer methods and costs of setting up and running the four electronic data capture systems

Features	Pendragon	CommCare	ODK Collect	MIVA
Integrated GPS	No	Yes	Yes	Yes
Inter-changeable language	No	Yes	Yes	Yes
Internet-free development	Yes	No	Yes	Yes
‘Cases’	No	Yes	No	No^a^
Logic				
Complexity of logic	Basic	Intermediate	Intermediate	Advanced
In-built analysis and feedback	Basic	Intermediate^b^	Intermediate^b^	Advanced
Data upload/download				
USB cable	Yes	No	Yes^c^	Yes
Internet	Yes	Yes	Yes	Yes
SMS	No	Yes	No	Yes
Cost				
Start-up costs	Medium	Low	Low	High
Running costs	Low	Medium	Low	Low

ODK: Open Data Kit; MIVA: Mobile InterVA (www.interva.net); Internet-free development: the system can be designed, tested and deployed in the field without needing to connect to the internet, assuming the necessary programmes are already downloaded; Start-up costs: includes purchasing hardware and person-time costs for programming; Running costs: includes phone charging allowances, data download costs and subscription fees.

a While the version of MIVA being used does not have this function, it has been added to the latest version.

b These EDC systems have simple calculated fields which can be displayed to the user or used in the form’s logic, allowing for basic in-built analysis and feedback, however MIVA uses a complex Bayesian model to analyse verbal autopsies into cause of death information.

c A programme called ODK Briefcase is used to download and amalgamate the data into.csv files.

Fieldworkers liked the integrated skip patterns as it simplified their interviews, and also reduced the amount of data cleaning required. Fieldworkers also commented on the ability to take photographs and videos as a positive feature in CommCare and ODK Collect. We are utilizing this feature as a data quality check, for example, photographing a child’s vaccination record, which we then compared to the recorded data to check the accuracy of data capture.

For CommCare and ODK Collect, there are additional online tools for data which has been submitted electronically. This includes the ability to view the data from any internet access point, create routine reports, and monitor fieldworker’s activity. We used this for one CommCare project, allowing the project manager to monitor progress while out of country, a very useful function when principal investigators are not always on site.

### Data processing and security

Although all EDC systems can submit data using an Internet connection (Table 2), this method was only used for CommCare in the current projects. Rather than putting SIM cards in fieldworkers’ phones, data were submitted when supervisors met interviewers, via local Wi-Fi networks set up on supervisors’ laptops using USB dongles connected to a cell phone network. The project manager noted that data could be transferred directly from the phone if fieldworkers were given airtime [phone credit] but this system is more open to abuse, where ‘abuse’ signifies using project phone credit for personal use. This was a driver in choosing USB downloading in the other EDC systems, an option not fully supported in CommCare. Pendragon fieldworkers reported problems during data download; this was due in part to damage to the PDAs’ USB ports from routine field use over 5 years and poor design of the connectors.

Security of data on the devices, often a concern with EDC, was not reported as an issue. Data stored by CommCare and ODK Collect are encrypted and the ‘apps’, as well as the smartphones can be password protected. Pendragon requires a password to download data. Data stored by MIVA can be encrypted, contains no identifying information, and cannot be interpreted without a translation process.

However, in one project we experienced SD card malfunctions as a result of purchasing poor quality SD cards locally, causing isolated data loss. We have also had two cases of SD cards being replaced or stolen while they were being charged at a commercial charging service. Of the 64 devices in the field, three devices have been stolen from fieldworkers’ houses in rural villages and three from a supervisor’s house in the district’s main town. Although we subsequently recovered two of these devices, the potential for data loss, the need to replace devices, and the security of fieldworkers needs to be planned for.

## Conclusion

Although PDAs and Pendragon were effective and cost-efficient in our case, as we already owned the hardware, this technology is becoming obsolete (although Pendragon has a version which runs on Android and iOS devices), and the software was harder to use than both CommCare and ODK Collect. We would not recommend purchasing PDAs over newer hardware, but if available, they still offer advantages over paper-based systems.

CommCare and ODK Collect have features that may make them appropriate for different contexts. ODK Collect is preferable in areas of poor Internet connectivity as the whole process from form development to data download can be done offline, and is well suited to cross-sectional surveys. CommCare is the appropriate software for studies or programmes requiring multiple encounters with the same respondents such as prospective cohorts, and our experience has shown that poor internet does not necessarily preclude CommCare from being used. In studies with one point of contact with respondents and reasonable internet connectivity, there is little to distinguish between CommCare and ODK Collect.

MIVA was programmed to perform a specific, standardised function, with all the required features built-in, making it very suitable for routine data collection. However, in a research setting we couldn’t customise it for our specific needs so we combined it with an ODK application, adding complexity to the interviewer’s task. Developing a stand-alone ‘app’ requires highly skilled people and more time, so we would not recommend this approach for small-scale research projects.

Our experience shows EDC to not only be viable, but desirable for data collection in a rural Sub-Saharan African setting, with each EDC system offering specific advantages and disadvantages. As we have described, the optimal hardware and software combination will be dependent on the nature of the project, budget, and local circumstances.
